# Beetroot Juice Enhances Nitrate Metabolism and Endothelial Function but Not Cardiovascular or Strength Performance in Bodybuilders with a History of Anabolic–Androgenic Steroid Abuse: A Crossover Trial

**DOI:** 10.3390/antiox15030321

**Published:** 2026-03-04

**Authors:** Leonardo Santos L. da Silva, Leonardo Da Silva Gonçalves, Marcio F. Tasinafo Junior, Yaritza B. Alves Sousa, Macario Arosti Rebelo, Carolina S. Guimaraes, Jose E. Tanus-Santos, Carlos R. Bueno Junior, Jonas Benjamim

**Affiliations:** 1 Department of Internal Medicine, Ribeirao Preto Medical School, University of São Paulo, Ribeirão Preto 14049-900, Brazilbuenojr@usp.br (C.R.B.J.); 2 Ribeirão Preto School of Physical Education and Sports, University of São Paulo, Ribeirão Preto 14049-900, Brazil; 3 Department of Health Sciences, Ribeirão Preto Medical School, University of São Paulo, Ribeirão Preto 14049-900, Brazil; 4 Department of Pharmacology, Faculty of Medical Sciences, State University of Campinas, Campinas 13083-875, Brazil; 5 Ribeirão Preto Nursing School, University of São Paulo, Ribeirão Preto 14049-900, Brazil; 6 Department of Pharmacology, Ribeirão Preto Medical School, University of São Paulo, Ribeirão Preto 14049-900, Brazil; tanus@fmrp.usp.br; 7 Institute for Physical Activity and Nutrition, School of Exercise and Nutrition Sciences, Deakin University, Geelong, VIC 3220, Australia

**Keywords:** nitrate, high blood pressure, cardiovascular risk, endothelial function, nitric oxide

## Abstract

Inorganic nitrate (NO_3_^−^) has demonstrated therapeutic efficacy in several populations characterised by cardiovascular risk. However, it is unknown whether increasing nitric oxide (NO) bioavailability affects vascular and cardiovascular responses in men with androgenic–anabolic steroid (AAS) abuse. Objective: To investigate the effects of dietary NO_3_^−^ on cardiovascular, autonomic, and strength performance in men with AAS abuse. Methods: In this double-blind, randomised, placebo-controlled crossover trial, participants consumed beetroot juice (12.8 mmol [800 mg] NO_3_^−^) or a placebo (0.3 mmol NO_3_^−^). After two hours, assessments included saliva collection, endothelial function, heart rate, and systolic (SBP) and diastolic (DBP) blood pressure at rest, during, and after an isometric handgrip test. Results: Thirteen resistance-trained males [mean (standard deviation) age: 31 (9) y; body mass index (BMI): 30 (4) kg/m^2^; SBP: 132 (3) mmHg; DBP: 70 (2) mmHg] completed the protocol. NO_3_^−^-rich juice significantly increased salivary NO_3_^−^ (40.6 μM, *p* < 0.001) and nitrite (NO_2_^−^) (3.1 μM, *p* = 0.002) versus placebo. Flow-mediated dilation was greater with NO_3_^−^ both at pre-exercise (2.37%, *p* = 0.02) and post-exercise (2.57%, *p* = 0.01). No between-group differences were observed in isometric strength (0.02 kgf, *p* = 0.99) or systolic/diastolic blood pressure across conditions. Conclusions: Dietary NO_3_^−^ enhanced salivary NO_2_^−^ and NO_3_^−^ concentrations and modestly improved endothelial function but did not reduce the elevated blood pressure or alter cardiac autonomic responses associated with AAS abuse.

## 1. Introduction

The abuse of androgenic–anabolic steroids (AAS) is associated with detrimental cardiovascular outcomes, primarily stemming from impairments in vascular function and dysregulation of cardiac autonomic control [1]. Among the notable cardiovascular alterations, epidemiological studies have elucidated transient arterial hypertension, autonomic dysfunction [2], and attenuated endothelial function [3,4]. These cardiovascular sequelae [5] are implicated in a threefold elevation in all-cause mortality risk, having cardiovascular adverse events as the primary leading cause [6]. While certain cardiac remodelling phenomena in habitual AAS users [7], such as hypertrophic cardiomyopathy, exhibit irreversible characteristics, recent findings have indicated that increased nitric oxide (NO) bioavailability can positively influence key independent cardiovascular risk factors [8,9,10] by decreasing blood pressure [11,12,13,14], restoring cardiac autonomic balance, and enhancing endothelial integrity in hypertension [15,16,17]. There is a growing body of literature that has substantiated the therapeutic efficacy of dietary inorganic nitrate (NO_3_^−^) within high-cardiovascular-risk (with hypertension and/or chronic heart failure) cohorts [18,19,20,21,22,23,24,25]. However, it is unclear whether increases in NO bioavailability can lead to improvements in cardiovascular variables in men with a history of androgenic–anabolic steroid abuse [26]. Accordingly, this study aimed to investigate the acute effects of inorganic NO_3_^−^ on cardiovascular responses at rest and during a standardised physical exertion in men with androgenic–anabolic steroid use. Based on the established role of NO_3_-derived NO in modulating vascular tone and metabolic efficiency, we hypothesised that NO_3_^−^ ingestion would improve endothelial function and cardiovascular responses during physiological stress. Conversely, we did not expect changes in maximal isometric strength performance, as dietary NO_3_^−^ primarily influences metabolic and vascular pathways rather than neural drive or motor unit recruitment, which are key determinants of maximal force production.

## 2. Materials and Methods

This randomised crossover study employed a double-masked and placebo-controlled design. The study protocol was approved by the Research Ethics Committee of the University of São Paulo, Brazil (CAAE: 66450622.4.0000.5659, 22 March 2023), and prospectively registered on ClinicalTrials.gov [NCT05835401] on 18 April 2023. All participants read and signed a consent form agreeing to the experimental protocol.

### 2.1. Participants

This study recruited adult men (18–45 years old) with current or previous ASS abuse for at least 12 months before inclusion. Participants were classified as current AAS users or previous AAS users based on self-reported history of anabolic-androgenic steroid use obtained through a structured interview. Previous AAS users had discontinued all AAS use, whereas current users maintained their habitual AAS use throughout the study period. Participants were excluded if they had a previous history of acute myocardial infarction/stroke, known allergy/intolerance to NO_3_^−^, or were currently under pharmacological therapy involving proton pump inhibitors, beta-blockers, calcium-antagonist channel, or antibiotic use over the last 4 weeks due to their potential influences on oral NO_3_^−^ reducing metabolism and function. During an initial screening, participants were asked about their health status and provided with a familiarisation session with research protocols. None of the enrolled participants had a clinical diagnosis of hypertension or were using antihypertensive drugs at the time of the study.

At the beginning of the session, blood samples were collected to analyse blood lipids and total free testosterone levels. In sequence, a dual X-ray absorptiometry was performed with the participants to analyse their body compartments (see Table 1).

### 2.2. Intervention

After the initial interview, participants were instructed to avoid foods high in NO_3_^−^ exceeding 15 mg per serving [27,28]. This guideline was applied throughout the study, including the washout period. Additionally, participants were advised not to use mouthwash, which is critical for maintaining oral microbiota NO_3_^−^ reducing capacity [29]. They completed a food questionnaire before each intervention and were asked to refrain from vigorous physical activity one day before and on testing days. One day before lab testing, participants were reminded not to consume caffeine (coffee, energy drinks, teas) for 12 h before each phase of the study and to avoid alcohol 24 h beforehand [30]. This study employed a two-arm intervention testing the effects of an acute dose of 140 mL of beetroot juice (BJ) rich in NO_3_^−^ 800 mg NO_3_^−^ [12.8 mmol]) compared to 140 mL of BJ depleted in NO_3_^−^ (0.3 mmol). The NO_3_^−^ content was based on manufacturer certification, with all products stored under recommended conditions, used within their expiration date, and functionally verified by increases in salivary NO_3_^−^ and NO_2_^−^ concentrations. Both interventions were matched in flavour, colour, and taste, which were purchased from the same manufacturer (James White Ltd., Ashbocking, UK). The randomisation (1:1) has been performed to allocate the participants to the first intervention using randomizer.org by an independent researcher who has not been involved with the research. After the first intervention, they completed a one-week washout and returned to perform the opposite intervention. The washout time was chosen based on previous studies that demonstrated that 7 days is enough to avoid carryover effects [21,31]. The process of concealing the treatment allocation order was performed by an independent researcher. Participants were not informed of the intervention order. The researcher measuring variables was blinded, while an external researcher handled the juice delivery. A masked collaborator conducted the statistical analysis.

### 2.3. Outcomes

The participants arrived in the laboratory in a fasted state (8 h) and then ingested the BJ. After a standard two-hour period, unstimulated saliva samples were collected using the spitting method [32] and quickly centrifuged at 3000 rpm at 4 °C for 15 min and stored at −80 °C. This was the only saliva collection in each condition. The laboratory environment has been controlled with a temperature between 22 and 24 °C and a humidity of 50–60% [15]. The evaluations started in the morning at the same time point (e.g., 07:00 a.m.) for each participant to standardise the influence of the circadian rhythm on the variables collected. During the evaluations, the participants kept quiet and silent [33].

#### 2.3.1. Blood Pressure

The sBP and dBP values were indirectly measured on the participant’s dominant arm using a clinically validated BP monitor (OMRON-M2^®^, HEM-7121-E, Sao Paulo, Brazil) previously calibrated. The measurements were performed in the supine position.

#### 2.3.2. Secondary Outcomes

**Figure 1:**
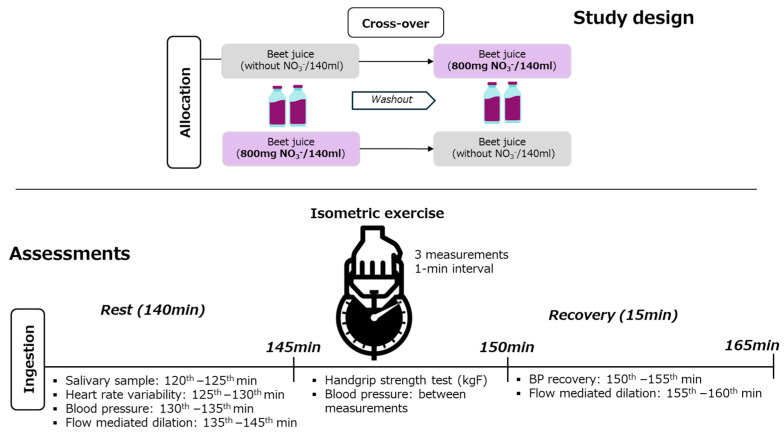
Study design and time point outcomes collection.

Heart rate (HR) was recorded beat-by-beat using a Polar^®^ H10 monitor (1000 Hz, RS800CX, Kempele, Finland). After each session, HR data were exported, and segments containing at least 256 stable R–R intervals (RRis) with more than 95% sinus beats were included. The initial 256 RRis from each 5 min window were imported into Kubios software (v2.1) to calculate HRV indices. Time-domain measures included the root mean square of successive differences (RMSSD) and the standard deviation of normalised R–R intervals (SDNN) [34,35,36,37]. Endothelial function was evaluated by flow-mediated dilatation (FMD) using a high-resolution Doppler ultrasound system 2D bidirectional ultrasound system (SAEVO^®^, Sao Paulo, Brazil) equipped with a 14 MHz linear transducer. Participants were positioned supine with the right arm abducted. The transducer was aligned longitudinally over the brachial artery, 5–10 cm above the antecubital crease, to measure pre-exercise diameter. The forearm cuff was subsequently inflated to 50 mmHg above the participant’s resting systolic blood pressure using an automated rapid-inflation system. The cuff remained inflated for five minutes to completely occlude blood flow to the forearm. After the 5 min occlusion period, the cuff was rapidly deflated to induce reactive hyperaemia. After cuff deflation, the brachial artery post-occlusion diameter was recorded for 2 min at the same site. Post-occlusion diameter was identified using manual frame-by-frame analysis by the same trained and experienced operator. Saliva was analysed for NO_2_^−^ and NO_3_^−^ concentration. An ozone-based reductive chemiluminescence assay has been performed, as previously described [38]. To quantify NO_2_^−^, 100 μL of saliva in duplicate was injected into an acidified tri-iodide solution purged with nitrogen and connected in-line with a chemiluminescence NO analyser (Sievers Model 280, Boulder, CO, USA). For NO_3_^−^ quantification, saliva samples were reduced with vanadium (III) in 1 mol/L HCl at 90 °C, and the released NO was carried by nitrogen gas to the analyser. In both assays, the reactive NO gas generated a chemiluminescent signal upon interaction with ozone (O_3_), and signal intensity was quantified using eDAQ-Chart software version 5.5.27 [38]. Isometric exercise test: To assess maximum voluntary contraction, a CAMRY^®^ brand adjustable and calibrated handgrip device with a scale of 0 to 100 kg was employed. Participants were seated with their shoulders slightly forward, elbows extended, arms beside the trunk and forearms and wrists in a neutral position. Their hand position was adjusted to the proximal interphalangeal joint positioned under the bar, and the grip was performed between the fingers and the thenar eminence with maximum comfort. During the test, participants performed a maximum of 5 s contractions for each arm with a 1 min interval [39,40]. Time points where each variable has been collected can be visualised in Figure 1.

### 2.4. Statistics

Our sample estimation was based on expected within-subject changes in systolic blood pressure (sBP) variation data from a prior meta-analysis investigating the NO_3_^−^ effectiveness after exercise [8]. Using the G*Power software (v. 3.1.9.2), a two-way ANOVA with repeated measures (three time points) was applied to assess the group-by-time interaction. The mean difference (SD) values of sBP following NO_3_^−^ supplementation were −4.5 (7) mmHg compared to placebo, yielding an estimated effect size of 0.46 [18]. To detect significant differences with 80% power (β = 0.2) at a significant level of 0.05, 12 participants were required. To account for potential dropouts, we aimed to recruit 14 participants. Data normality distribution was assessed using the Shapiro–Wilk test. Statistical analyses were conducted with RStudio (v. 5.0.0) using an intention-to-treat approach. A linear mixed model (LMM) evaluated the effectiveness of the NO_3_^−^ intervention versus placebo, accounting for participants as a random effect, along with time and treatment group interactions. Interventions were treated as a group factor, time points from the submaximal exercise test and recovery were treated as repeated measures (the resting values of each variable have been used as covariates in the final model). The effects of treatments on cardiovascular measurements before and after exercise were tested, applying Bonferroni’s correction for multiple comparisons. Intergroup differences (placebo vs. NO_3_^−^) were assessed using LMM in direct comparisons, or *t*-test or Mann–Whitney U test, with significance set at *p* < 0.05.

## 3. Results

**Figure 2:**
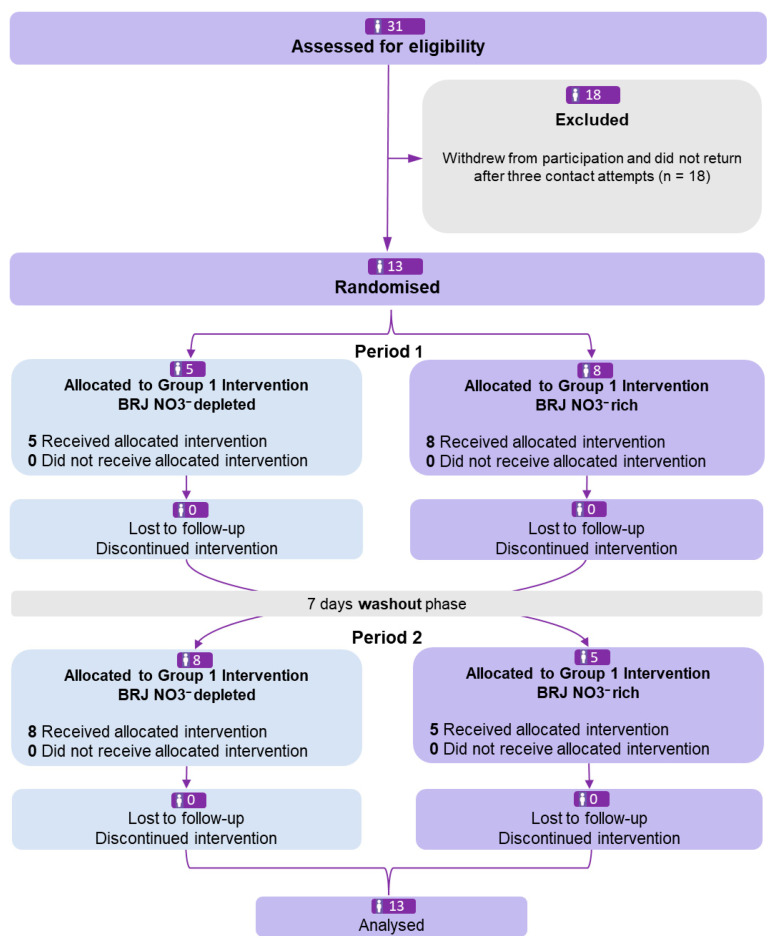
CONSORT flowchart.

Thirteen apparently healthy resistance-trained men under current (~77%) and previous AAS use (~23%) for at least 12 months completed the randomised, crossover trial without dropouts (Figure 2). All details of the drugs used by the sample are presented in the Supplementary Material Table S1.

**Figure 3:**
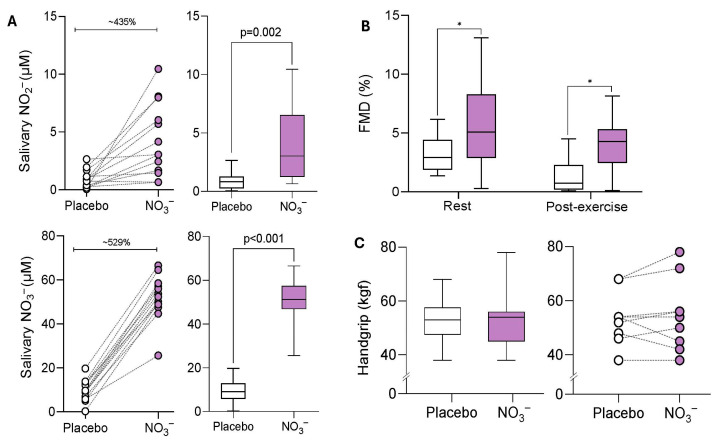
Effects of NO_3_^−^ from beetroot juice on saliva NO_3_^−^ (μM), NO_2_^−^ (μM) concentration and flow-mediated dilation (%) and handgrip isometric strength test (kgf) outcomes. μM: micromol; kgf: kilogram-force. (**A**) Differences between intergroup conditions in saliva NO_3_^−^ and NO_2_^−^ concentrations were analysed using a *t*-test or Mann–Whitney (non-parametric approach) as appropriate, * *p* < 0.05. (**B**) Intergroup comparisons (placebo vs. nitrate) to flow-mediated dilation were performed using a linear mixed model (* *p* < 0.05). (**C**) Intergroup comparison (placebo vs. nitrate) to handgrip test was performed using a linear mixed model (* *p* < 0.05). White: Placebo; Purple: Beetroot juice rich in NO_3_^−^.

Supplementation with NO_3_^−^-rich BJ effectively increased salivary NO_3_^−^ (40.59 μM [95%CI: 34.99 to 46.19], *p* < 0.001) and NO_2_^−^ (3.08 μM [95%CI: 1.26 to 4.90], *p* = 0.002) concentrations compared with the placebo, confirming protocol compliance (Figure 3A). Endothelial function (flow-mediated dilation) was significantly greater in the NO_3_^−^ group compared with placebo under both pre-exercise (2.37% [95%CI: 0.31 to 4.40], *p* = 0.02) and post-exercise conditions (2.57% [95%CI: 0.54 to 4.60], *p*= 0.01). These findings demonstrated that NO_3_^−^ supplementation enhanced endothelial function independently of exercise (Figure 3B). Comparisons between protocols (NO_3_^−^ vs. placebo) to the isometric strength test (handgrip) did not reveal changes (0.02 kgf [95%CI: −9.74 to 9.71, *p* = 0.99) (Figure 3C).

**Figure 4:**
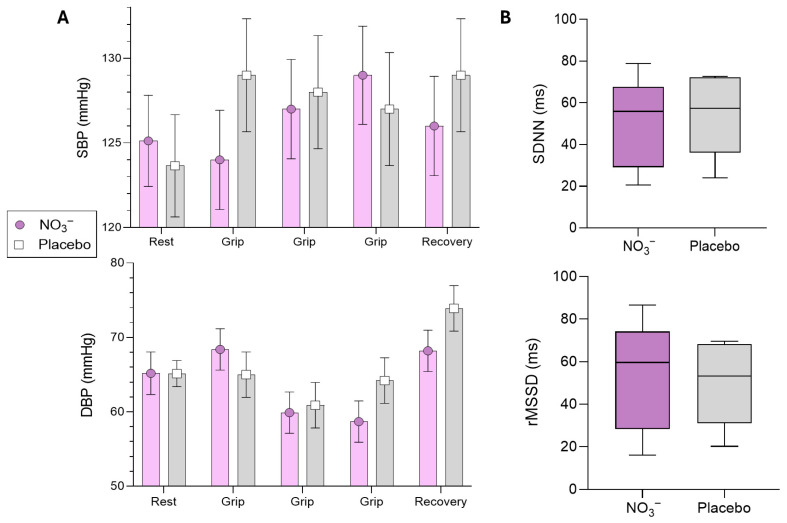
Effects of NO_3_^−^ from beetroot juice on blood pressure and HRV indices. SDNN: standard deviation of R–R normalised (NN) intervals. RMSSD: root mean square of R–R intervals, successive differences. mmHg: millimetres of mercury; ms: milliseconds. (**A**) Differences between intergroup conditions comparison (placebo vs. nitrate) to blood pressure were performed using a linear mixed model. (**B**) SDNN and RMSSD and handgrip strength test were analysed with *t*-test or Mann–Whitney (non-parametric approach) as appropriate. Data is presented as mean (SD).

Regarding blood pressure responses, the sBP showed no significant differences between conditions during the exertion (Grip 1: NO_3_^−^ vs. PLA: −4.0 mmHg [95%CI: −11.2 to 3.20, *p* = 0.270]; Grip 2: NO_3_^−^ vs. PLA: −0.7 mmHg [95%CI: −7.8 to 6.5, *p* = 0.858]; and Grip 3: NO_3_^−^ vs. PLA: −1.3 mmHg [95%CI: −5.9 to 8.4, *p* = 0.731]) and during recovery from exercise (NO_3_^−^ vs. PLA: −2.4 mmHg [95%CI: −9.6 to 4.8, *p* = 0.506]). Similarly, dBP also did not show significant differences between conditions during the exertion (Grip 1: NO_3_^−^ vs. PLA: 3.4 mmHg [95%CI: −3.5 to 10.3, *p* = 0.326]; Grip 2: NO_3_^−^ vs. PLA: −1.0 mmHg [95%CI: −7.9 to 5.9, *p* = 0.768]; and Grip 3: NO_3_^−^ vs. PLA: −5.6 mmHg [95%CI: −12.4 to 1.3, *p* = 0.112]) and during recovery from exercise (NO_3_^−^ vs. PLA: −5.7 mmHg [95%CI: −12.5 to 1.2, *p* = 0.104]) (Figure 4A). In addition, no changes were found to HRV indices, SDNN (*p* = 0.99) or RMSSD (*p* = 0.68), suggesting that NO_3_^−^ was unable to enhance cardiac autonomic modulation at rest (Figure 4B).

## 4. Discussion

To our knowledge, this is the first study to investigate the effects of dietary NO_3_^−^ in men undergoing AAS abuse. Our preliminary findings indicated that enterosalivary pathway metabolism has been changed upon NO_3_^−^ ingestion, observed by an increase in saliva NO_3_^−^ and NO_2_^−^. In this regard, we also observed that some participants blunted the oral capacity to reduce NO_3_^−^ orally, being noted by no individual changes in saliva NO_2_^−^ concentrations across the dietary NO_3_^−^ and placebo conditions. Our findings also demonstrated that NO_3_^−^ can elicit changes in endothelial functions assessed by FMD aligned with increases in oral NO_3_^−^/NO_2_^−^ concentrations following NO_3_^−^ ingestion, but these improvements did not translate to changes in blood pressure, even at rest and during post-exercise. Based on these findings, the detrimental effects of AAS on blood pressure and on cardiac autonomic modulation may not be counteracted with increased NO bioavailability.

The clinical relevance of the observed improvement in FMD should be interpreted within the acute nature of the intervention. Acute NO_3_^−^ studies consistently report modest but significant increases in FMD, reflecting enhanced endothelial NO-dependent responsiveness rather than long-term vascular adaptation. The magnitude of the FMD change observed in the present study is comparable to that reported in prior acute interventions and falls within a range generally considered physiologically meaningful in endothelial function research [17,41]. However, while such acute improvements are indicative of favourable vascular responsiveness, they should not be interpreted as direct evidence of sustained cardiovascular risk reduction. Notably, some participants appeared to exhibit a blunted oral NO_3_^−^-reducing capacity, evidenced by the absence of a rise in salivary NO_2_^−^ after NO_3_^−^ ingestion. This inter-individual variability is likely driven primarily by differences in the oral microbiome, particularly the abundance and activity of NO_3_^−^-reducing bacteria located on the tongue [42,43,44]. Recent oral hygiene practices, such as toothbrushing, tongue scraping, or the use of antibacterial mouthwash, may acutely suppress these bacteria and impair NO_3_^−^ to NO_2_^−^ conversion. Habitual diet may also play a role, as regular intake of NO_3_^−^-rich vegetables may promote microbial adaptation, whereas low plant consumption may reduce this capacity [45]. Additional contributors include salivary flow rate, circadian variation, smoking, medication use (e.g., antibiotics or proton pump inhibitors), ageing, and cardiometabolic health [46,47]. Together, these factors highlight the strong oral and lifestyle dependence of the enterosalivary NO_3_^−^-NO_2_^−^-NO pathway and help explain the variability in salivary NO_2_^−^ responses.

The participants in our sample presented high blood pressure levels but lacked a hypertension clinical diagnosis, and the lack of effects on this variable indicates that AAS abuse can target other mechanisms (e.g., oxidative stress) related to blood pressure not assessed in this study [48,49]. The handgrip test has been used to induce cardiovascular stress in this population and mimic isometric strength training routinely performed by this population in a gymnasium. As expected, no changes have been identified between NO_3_^−^ and placebo conditions. The lack of effects during the handgrip strength test is strongly supported by previous research [28]. Furthermore, these outcomes are widely explained by the bioenergetics contributions to NO_3_^−^ to exercise capacity, where it is more related to exercise actions that last between 2 and 10 minutes of duration [50,51]. During the initial six months of recruitment, several participants gave up participating. It delayed the conclusion of the research, but most importantly, it shed light on the challenges to developing research projects focused on health in this population. Importantly, the small sample size and the absence of a non-AAS control group limit the generalizability of the present findings, and therefore, the results should be interpreted with caution. Due to the exploratory nature of this study, we would be pleased to see further randomised trials on this topic to confirm these results, but also to dive into variables not assessed in our research.

For instance, our findings demonstrate that while dietary inorganic NO_3_^−^ can enhance oral NO_3_^−^ metabolism through NO_3_^−^ and NO_2_^−^ salivary concentrations and elicit modest improvements in endothelial function, acutely these adaptations do not mitigate the persistent elevations in blood pressure induced by AAS abuse or improvements in cardiac autonomic modulation. It suggests that alternative or complementary mechanisms beyond nitric oxide bioavailability may underlie the blood pressure dysregulation observed in this population (e.g., oxidative stress, baroreflex impairment, microvascular rarefaction). However, due to the nature of the study (acute) and limited sample size, future long-term clinical trials with this population are warranted.

Previous studies investigating NO_3_^−^ supplementation have consistently demonstrated improvements in endothelial function, particularly FMD, whereas effects on autonomic modulation and neuromuscular performance appear more variable and context-dependent [17]. Acute NO_3_^−^ ingestion has been shown to enhance FMD in healthy and clinical populations, supporting the notion that endothelial responsiveness is especially sensitive to short-term increases in NO bioavailability. In contrast, changes in HRV are less consistently observed following acute supplementation and may require longer intervention periods, higher training stimuli, or populations with greater baseline impairment [35,52]. Key limitations include the short supplementation duration and limited power for secondary outcomes. Future studies should consider longer-term NO_3_^−^ supplementation, larger samples, and the inclusion of additional vascular (e.g., arterial stiffness, microvascular reactivity), autonomic (e.g., baroreflex sensitivity), and neuromuscular or performance-related outcomes to better delineate the temporal and mechanistic effects of inorganic NO_3_^−^.

## Figures and Tables

**Table 1:** Characteristics of the participants are presented in mean values followed by their respective standard deviations (SDs) of age, height, body mass, BMI (kg/m^2^), fat-free mass, fat mass, visceral fat, bone mineral content, total free testosterone, lipid profile, resistance training history, and profile of AAS drug abuse.

Variables (n = 13)	Values
Age (years)	31 (9)
Height (m)	1.74 (0.05)
Body mass (kg)	93.1 (10)
BMI (kg/m^2^)	30 (4)
Fat-free mass (kg)	74.9 (11)
Body fat mass (%)	16.9 (5.6)
Visceral fat (kg)	0.5 (0.2)
Bone mineral content (kg)	3.5 (0.4)
Total free testosterone (ng/dL)	1118 (495)
Total cholesterol (mmol/L)	4.95 (1.43)
HDL (mmol/L)	0.91 (0.3)
LDL (mmol/L)	3.7 (1.36)
TGL (mmol/L)	1.87 (0.23)
SBP (mmHg)	132 (3)
DBP (mmHg)	70 (2)
**Resistance training history**	
Training time (years)	11.6 (4.8)
Current training (sessions/week)	5.3 (1.2)
**History of AAS use**	
Drugs used in one cycle (%)	
<3	25
3–5	75
>5	0
**Most used drugs in one cycle (%)**	
Injectable	75
Oral	12.5
Both combined	12.5
**Number of cycles performed throughout life (%)**	
1–3	50
4–5	37.5
>5	12.5
**AAS use status (%)**	
Current	76.9
Previous	23.1
**Purpose of using AAS (%)**	
Competitive (bodybuilding)	46.1
Aesthetic/recreational	53.9

m: metres; kg: kilograms; ng/dL: nanograms per decilitre; mmol/dL: millimole per decilitre; mmHg: millimetres of mercury; HDL: high-density lipoprotein; LDL: low-density lipoprotein; TGL: triglycerides; SBP: systolic blood pressure; DBP: diastolic blood pressure.

## Data Availability

The original contributions presented in this study are included in the article/Supplementary Material. Further inquiries can be directed to the corresponding author.

## Supplementary Materials

The following supporting information can be downloaded at https://www.mdpi.com/article/10.3390/antiox15030321/s1.

## Abbreviations

The following abbreviations are used in this manuscript:

BMI	Body mass index
BP	Blood pressure
BJ	Beetroot juice
dBP	Diastolic blood pressure
DXA	Dual x-ray absorptiometry
FFM	Fat-free mass
FMD	Flow-mediated dilation
HDL	High-density lipoprotein)
HR	Heart rate
HRV	Heart rate variability
ITT	Intention to treat approach
kg	kilogram
LDL	Low-density lipoprotein
LMM	Linear mixed models
m	Metres
mg	Milligram
min	Minutes
mL	Millilitre
mmHg	Millimetre of mercury
NO_2_^−^	Nitrite
NO_3_^−^	Nitrate
NOS	Nitric oxide synthesis
sBP	Systolic blood pressure
SD	Standard deviation
TGL	Triglyceride
