# Supplementary material for: Beetroot Juice Enhances Nitrate Metabolism and Endothelial Function but Not Cardiovascular or Strength Performance in Bodybuilders with a History of Anabolic–Androgenic Steroid Abuse: A Crossover Trial
Source: Antioxidants (Basel). 2026 Mar 4;15(3):321. doi: 10.3390/antiox15030321 (PMC13024152; doi:10.3390/antiox15030321)
Supplement: Supplementary file 1 [file antioxidants-15-00321-s001.zip › antioxidants-4161755-supplementary.pdf]

Supplementary Table S1. Frequency and percentage of anabolic-androgenic steroids used by compounds among participants.

Drug	Percentage of users (%)
Oral	
Fluoxymesterone (Halotestin)	12.5
Oxandrolone	62.5
Oxymetholone (Hemogenin)	50
Stanozolol	50
Ethylestrenol	0
Methandrostenolone (Dianabol)	75
Methenolone acetate	12.5
Testosterone undecanoate	0
Quinbolone	0
Methylandrostenediol (Metandriol)	0
Norethandrolone	0
4-Chlorodehydroethyltestosterone	0
Mesterolone (Proviron)	37.5
Injectable	
Nandrolone decanoate (Deca)	75
Nandrolone phenylpropionate (NPP)	25
Nandrolone cypionate	0
Nandrolone hexylphenylpropionate	0
Nandrolone undecanoate	0
Nandrolone laurate	0
Nandrolone cyclohexylpropionate	0
Testosterone enanthate	87.5
Testosterone cypionate (Deposteron)	62.5
Testosterone propionate	62.5
Testosterone cyclohexylpropionate	0
Testosterone phenylpropionate	25
Drostanolone propionate (Masteron)	62.5
Methenolone enanthate (Primobolan)	12.5
Methylandrostenediol	0
Trenbolone hexahydrobenzylcarbonate (Parabolan)	0
Trenbolone acetate	62.5
Oxabolone	0
Stanozolol (injectable)	50
Testosterone blend (Sustanon-type)	75
Boldenone	50
